# Transoral Drug-Induced Sleep Endoscopy: A Useful Complementary Tool in Sleep Surgery

**DOI:** 10.1055/s-0044-1788768

**Published:** 2025-01-10

**Authors:** Ahmed Elsobki, Mohammed Elshaer, Hassan Ghabn, Mohamed E. El-deeb, Luci Suliman

**Affiliations:** 1 Department of Otorhinolaryngology, Faculty of Medicine, Mansoura University, Mansoura, Egypt; 2 Department of Otorhinolaryngology, Faculty of Medicine, Kafrelsheikh University, Kafrelsheikh, Egypt; 3 Department of Chest Diseases, Faculty of Medicine, Mansoura University, Mansoura, Egypt

**Keywords:** sleep apnea syndromes, snoring, CPAP, tongue, polysomnography

## Abstract

**Introduction** Drug-induced sleep endoscopy (DISE) is performed widely, and several studies have demonstrated its validity as it provides clinical information not available by routine clinical inspection alone.

**Objective** This study aims to evaluate the role of transoral drug-induced sleep endoscopy (DISE) in the evaluation of tongue-palate (TP) interaction and its impact on surgical outcomes.

**Methods** A total of 42 patients with known obstructive sleep apnea syndrome (OSAS) were classified into two groups according to TP interaction (the absence of space between tongue and palate with the visual impression that the tongue is pushing the soft palate) into +ve and –ve TP interaction. Snoring according to the visual analogue scale (VAS), the Epworth Sleepiness Scale (ESS), and sleep study data were recorded before and after the pharyngoplasty operation.

**Results** There was a statistically significant difference between studied groups postoperative regarding minimal oxygen saturation, snoring index, apnea-hypopnea index (AHI), the ESS, and visual analogue scale of snoring ( *p* = 0.003*, *p* < 0.001*, *p* < 0.001*, *p* = 0.004*, and *p* = 0.003*, respectively). It displayed a marked higher average improvement among cases with –ve than in those with +ve TP interaction in terms of snoring index, AHI, and ESS.

**Conclusion** The Transoral DISE Has A Valuable Role In Evaluating And Assessing TP Interaction And Its Importance On Surgical Outcomes. Cases With Positive TP Interaction Show Poor Response To Isolated Palatopharyngeal Expansion And Need Further Analysis To Create A Better Treatment Plan And Improve Outcomes.

## Introduction

Obstructive sleep apnea (OSA) is a breathing disorder that occurs during sleep, is characterized by frequent attacks of upper airway collapse, with a subsequent reduction (hypopnea) or absence (apnea) in airflow lasting for at least 10 seconds and accompanied by either cortical arousal or a fall in blood SPO _2_ . 1

The pathophysiology of this syndrome is complex and multifactorial. Several factors influence the stability of the pharynx and might contribute to its closure during sleep when the activity of the pharyngeal dilating muscles is considerably reduced. 2

Anatomically, the dorsum of the tongue is anterior to the soft palate. Based on the observation of posterior displacement of the tongue during obstructive apnea, Isono et al. hypothesized that the dorsum of the tongue pushes the anterior wall of the soft palate posteriorly during inspiratory efforts, maintaining closure of the retropalatal airway. On the other hand, they evaluated the dynamic interaction between the tongue and the soft palate during OSA by manometric studies on 14 patients with sleep-disordered breathing. They found close apposition between the tongue and soft palate in all patients, and a progressive increase in contact pressure during OSA. 3

Drug-induced sleep endoscopy (DISE) is widely performed, and several studies have demonstrated its validity; in fact, it provides clinical information not available by routine clinical inspection alone. 4 Another diagnostic tool that is useful in conjunction with the standard DISE is the transoral fiber-optic endoscopy, which could give additional information in selected patients if the mouth is open. 5

Our study aims to assess the role of transoral drug-induced sleep endoscopy in the evaluation of tongue-palate (TP) interaction (the absence of space between tongue and palate with the visual impression that the tongue is pushing the soft palate), and its impact on surgical outcomes in such patients.

## Methods

This study included 42 patients, 24 males (57.1%) and 18 females (42.9%), and their ages ranged between 25 and 54-years-old. This research was performed in the university hospitals from August 2019 to December 2020 and approved by the university's ethical committee with the code MS/19.07.719. Informed written consent was taken from all patients after explanation.

### Inclusion Criteria

We included adults over 18-years-old, with OSA syndrome, fit for general anesthesia, having failed or refused CPAP therapy, with negative tongue collapse according to the transnasal DISE.

### Exclusion Criteria

The exclusion criteria were patients unfit for general anesthesia, those with previous sleep surgeries, cases with tongue base and laryngeal collapse according to the transnasal DISE, and cases with craniofacial anomalies.

### Evaluation

A thorough medical history was obtained using a visual analogue scale (VAS) for snoring 6 and the Arabic version of the Epworth Sleepiness Scale (ESS). 7 A general examination was performed concerning body mass index (BMI) and neck circumference. Additionally, an ENT examination was performed for a deviated septum, hypertrophied turbinates, the Friedman tongue position (FTP), as well as the oral cavity for the tongue, uvulae, tonsils (Brodsky scale, in five grades), and the relationship between the tongue and soft palate to determine whether an enlarged tongue obscures the vision of the palate.

### Polysomnography

The polysomnography examination was performed preoperatively and 6 months postoperatively, utilizing the SOMNOscreen plus (SOMNOmedics AG, Randersacker, Germany) device to diagnose and evaluate the degree of OSAS by a chest consultant (the last author). Apnea and hypopnea were the main obstructive events in OSA, with apnea being described as the stoppage of airflow at the nostrils and mouth for at least 10 seconds, and hypopnea as a decrease of more than 50% in oronasal airflow for at least 10 seconds accompanied by more than 4% decrease in oxyhemoglobin saturation from baseline level and/or arousal. 8

### Drug-Induced Sleep Endoscopy (DISE)

Patients were placed in a supine position with basic cardiorespiratory monitoring. The required depth of sedation was to reach the unconscious state, in which the subject started to snore.

Sleep was induced using propofol in a 1.5 mg/kg dose as a bolus and then maintained with the simple manual controlled infusion. This drug allowed higher sedation throughout the process of endoscopy. 9 Greater degrees of sedation were accompanied by a marked decrease in upper airway dilator muscle tone and neuromuscular reflex stimulation, raising airway collapsibility and avoiding oversedation during DISE. We did not record or analyze the collapse that happened while oxygen saturation was lower than the saturation nadir in PSG.

**Fig. 1:**
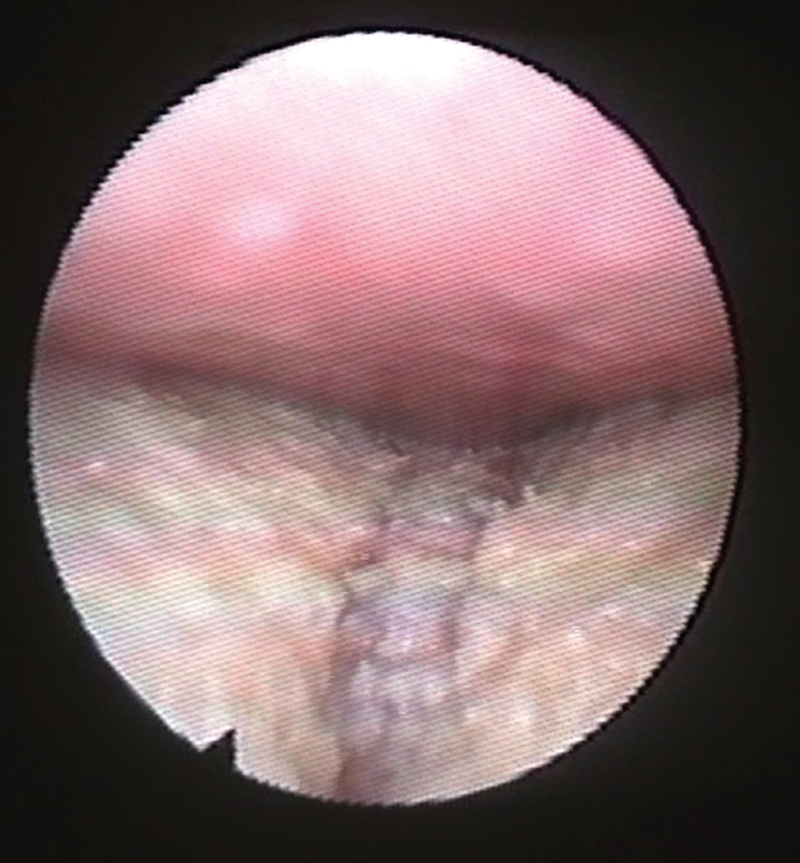
Endoscopic imaging showing ( **A** ) positive and ( **B** ) negative TP interactions.

A transnasal DISE was performed, with a flexible endoscope lubricated with lidocaine gel being introduced to the nasal cavity. The observation was conducted according to the lateral pharyngeal wall, palate, tongue, and larynx (LwPTL) classification, where the sites generating snoring and obstruction were assessed. Then, a transoral DISE was performed as the endoscopy was smoothly pushed among both incisors without the need to force the mouth open, to evade the effect of mouth breathing on the upper airway structure. Positive TP interaction was defined as the absence of space between tongue and palate with the visual impression that the tongue pushed the soft palate, 10 as shown in Figure 1 .

### Surgical Technique

The updated Cahali lateral pharyngoplasty 11 was performed in all cases, relying mainly on a palatopharyngeus muscle flap by the senior author. 12

### Follow-up

All patients were postoperatively checked at 1-week, 2-weeks, 1-month, and 6-months as outpatient visits to assess any complications and manifestation relief in terms of snoring according to the visual analogue scale and sleepiness according to the ESS. Additionally, polysomnography was performed 6 months postoperatively to calculate the apnea–hypopnea index (AHI) and other respiratory parameters to compare it to the preoperative data. Finally, the Sher success criteria were used to define the surgical success of the operation; 50% reduction of the preoperative AHI and of the < 20 events/hour. 13

### Statistical Analysis

Data were fed to the computer and analyzed using the Statistical Package Social Sciences (SPSS, IBM Corp., Armonk, NY, USA), version 22.0 for Windows. Qualitative data were described using numbers and percentages. Quantitative data were described using the mean and standard deviation (SD) for parametric data after testing normality with the Kolmogorov–Smirnov test. The significance of the obtained results was judged at the 0.05 level, and a highly significant difference was present if *p* ≤ 0.001. The qualitative data analysis was performed using the Chi-squared test to compare two or more groups, and the Monte Carlo test as correction when more than 25% of cells have to count less than 5 in tables (>2*2). Data analysis of quantitative data was performed using a Student *t* -test to compare two independent groups, while the paired *t* -test was to compare two studied periods. We used the Z test for comparing proportions. Binary stepwise logistic regression analysis was used for the prediction of independent variables with a binary outcome. Significant predictors in the univariate analysis were entered into the regression model using the forward Wald method. Adjusted odds ratio (OR) and their 95% confidence interval (CI) were calculated.

## Results

This study included 42 patients, 24 males (57.1%) and 18 females (42.9%), and the mean age of the studied cases was 38.95 ± 6.99, with a mean BMI of 32.13, ranging from 26 to 39.1 kg/m ^2^ . There is no statistically significant age difference between the –ve and +ve TP interaction groups). However, there is a statistically significant higher TP interaction among females and those with a higher BMI ( Table 1 ).

**Table 1:** Sociodemographic characteristics of the two studied groups

	-ve TP interaction n = 36	+ve TP interaction n = 6	Test of significance
Age, years (mean ± SD)	38.58 ± 7.14	41.17 ± 6.11	*t* = 0.835 *p* = 0.409
**Sex, n (%)**	
Female	13 (36.1)	5 (83.3)	χ ^2^ = 4.7
Male	23 (63.9)	1 (16.7)	*p* = 0.03*
BMI (kg/m ^2^ ), mean ± SD	30.94 ± 3.87	35.25 ± 2.69	*t* = 2.61 *p* = 0.012*

**Abbreviations:** BMI, body mass index; SD, standard deviation; TP, tongue-palate; t, Student *t* -test. **Notes:** *statistically significant if *p* < 0.05.

There is no statistically significant difference between studied groups regarding minimal SpO _2_ preoperative, while there is a statistically significant difference between both groups postoperative with a higher percentage of improvement among –ve than the +ve TP interaction groups (11.65 vs. 9.7%, respectively). Also, there is no statistically significant difference between studied groups regarding snoring index preoperative, while there is a statistically significant difference between studied groups postoperative with a higher percentage of improvement among –ve than the +ve TP interaction groups (48.8 vs. 25.2%, respectively), as shown in Table 2 .

**Table 2:** Sleep parameters before and after surgery among the studied groups

	-ve TP interaction n = 36	+ve TP interaction n = 6	Test of significance
Minimal preoperative SpO _2_ (mean ± SD)	78.69 ± 5.59	75.50 ± 4.76	*t* = 1.32 *p* = 0.195
Minimal postoperative SpO _2_ (mean ± SD)	87.86 ± 3.76	82.83 ± 2.48	*t* = 3.15 *p* = 0.003*
Paired *t* -test	t = 14.17 *p* < 0.001*	t = 4.03 *p* = 0.01*	
Percent of change	11.65%	9.7%	
Preoperative snoring index (mean ± SD)	157.0 ± 35.36	184.67 ± 18.78	*t* = 1.86 *p* = 0.07
Postoperative snoring index (mean ± SD)	80.33 ± 32.14	138.17 ± 18.71	*t* = 4.26 *p* < 0.001*
Paired *t* -test	t = 19.43 *p* < 0.001*	t = 4.98 *p* = 0.004*	
Percent of change	48.8%	25.2%	
Preoperative AHI (mean ± SD)	33.75 ± 11.74	46.68 ± 8.72	*t* = 2.57 *p* = 0.014*
Postoperative AHI (mean ± SD)	15.94 ± 8.40	33.0 ± 4.60	*t* = 4.82 *p* < 0.001*
Paired *t* -test	t = 14.0 *p* < 0.001*	t = 7.31 *p* = 0.001*	
Percent of change	52.8%	29.3%	
Preoperative ESS (mean ± SD)	18.08 ± 3.90	20.33 ± 3.56	*t* = 1.32 *p* = 0.194
Postoperative ESS (mean ± SD)	12.17 ± 3.49	17.0 ± 4.05	*t* = 3.07 *p* = 0.004*
Paired *t* -test	t = 11.76 *p* < 0.001*	t = 3.78 *p* = 0.013*	
Percent of change	32.7%	16.4%	
Preoperative VAS (mean ± SD)	7.5 ± 2.1	8.2 ± 1.9	*t* = 0.765 *p* = 0.449
Postoperative VAS (mean ± SD)	3.24 ± 0.58	4.5 ± 2.1	*t* = 3.11 *p* = 0.003*
Paired *t* -test	t = 2.5 *p* < 0.001*	t = 3.1 *p* < 0.001*	
Percent of change	56.8	45.1	

**Abbeviations:** AHI, apnea-hypopnea index; ESS, Epworth Sleepiness Scale; SD, standard deviation; SpO _2_ , oxygen saturation; t, Student *t* -test; VAS, visual analogue scale.

Note: *statistically significant if *p* < 0.05.

There is a statistically significant difference between the studied groups regarding the AHI preoperative. While there is a statistically significant difference between both groups postoperative with a higher percentage of improvement among the –ve compared to the +ve TP interaction group (52.8 vs. 29.3%, respectively). There is a statistically significant difference in the ESS between studied groups after surgery without significant difference preoperative. A higher percentage of improvement among the –ve (32.7%) compared with the +ve (16.4%) TP interaction groups was detected ( Table 2 ).

There is a statistically significant difference in VAS score between studied groups postoperative without significant difference preoperative with a higher percentage of change among the group with –ve TP interaction (56.8 vs. 45.1%, respectively). ( Table 2 )

Binary stepwise logistic regression analysis illustrates that preoperative AHI, BMI, sex, and preoperative snoring index have no statistically significant effect on TP interaction ( Table 3 ).

**Table 3:** Multivariate analysis of confounding factors of TP interaction

	B	SE	Wald	DF	*p* -value	OR	95% CI for OR	
							Lower	Upper
Preoperative AHI	0.114	0.081	1.993	1	0.158	1.121	0.957	1.314
BMI	−0.017	0.138	0.014	1	0.905	0.984	0.751	1.288
Sex	0.225	1.078	0.044	1	0.835	1.252	0.151	10.354
Preoperative snoring index	0.001	0.031	0.000	1	0.987	1.001	0.942	1.063
Constant	−6.272	5.791	1.173	1	0.279	0.002		

**Abbreviations:** AHI, apnea-hypopnea index; B, unstandardized regression weight; BMI, body mass index; CI, confidence interval; DF, degree of freedom; OR, odds ratio; SE, standard error; TP, tongue-palate.

There is a statistically significant higher percentage of overall success among the group with negative TP interaction than the one with positive interaction (72.2% vs. zero). However, a nonstatistically significant difference is detected between negative and positive TP interaction as regards snoring index, minimal SpO _2_ , AHI, and ESS percentage of improvement ( Table 4 ).

**Table 4:** Percentage of improvement in snoring index, minimal SpO _2_ , AHI, ESS among studied groups

Percentage of improvement (%)	-ve TP interaction n = 36	+ve TP interaction n = 6	Test of significance (z test)
Snoring index	48.8	25.2	z = 1.07 *p* = 0.282
Minimal SpO _2_	11.65	9.7	z = 0.14 *p* = 0.889
AHI	52.8	29.3	z = 1.07 *p* = 0.287
ESS	32.7	16.4	z = 0.80 *p* = 0.422
Overall success	72.2	0.0	z = 3.36 *p* = 0.001*

**Abbreviations:** AHI, apnea-hypopnea index; ESS, Epworth Sleepiness Scale; SpO _2_ , oxygen saturation; TP, tongue-palate. **Note:** *statistically significant if *p* < 0.05

Table 5 showed that there was a statistically significant difference between studied groups regarding DISE palate classification with 66.7% of the cases with +ve TP interaction having low palatal collapse, compared with 58.3% of the cases with –ve.

**Table 5:** The DISE distribution among the studied groups

	-ve TP interaction n = 36 (%)	+ve TP interaction n = 6 (%)	Test of significance
**DISE larynx**			FET *p* = 1.000
**L0**	36 (100)	6 (100)	
**L1**	0 (0.0)	0 (0.0)	
**DISE tongue**			MC *p* = 1.000
**T0**	36 (100)	6 (100)	
**TH**	0 (0.0)	0 (0.0)	
**TL**	0 (0.0)	0 (0.0)	
**DISE palate**			MC *p* = 0.001*
**P0**	15 (41.7)	0 (0.0)	
**PHL**	0 (0.0)	2 (33.3)	
**PL**	21 (58.3)	4 (66.7)	
**DISE lateral wall**			MC *p* = 0.478
**LH**	4 (11.1)	0 (0.0)	
**LS**	5 (13.9)	0 (0.0)	
**LV**	25 (69.4)	6 (100)	
**LW0**	2 (5.6)	0 (0.0)	

**Abbreviations:** DISE, drug-induced sleep endoscopy; FET, Fischer exact test; L0, no laryngeal collapse; L1, laryngeal collapse; LH, lateral wall at hypopharynx; LS, lateral wall at salpingopharyngeal folds; LV, lateral wall at the velum; LW0, no collapse at the lateral wall; MC, Monte Carlo test; P0, no palatal collapse; PHL, high palate collapse of the vertical palate; PL, low palate collapse of the oblique palate; T0, no tongue collapse; TH, high tongue base collapse; TL, low tongue base collapse; TP, tongue-palate. **Note:** *statistically significant.

In our study, the Friedman tongue position (FTP) in the +ve TP interaction group was grade III in 1 patient, II in 3 patients, and I in 2.

There were 2 cases (4.76%) with secondary bleeding and 2 (4.76%) with velopharyngeal insufficiency in terms of complications.

## Discussion

The current was a prospective interventional study conducted on 42 cases who underwent transoral sleep endoscopy, demonstrating that 6 cases had +ve and 36 had -ve TP interaction. The cases were operated on by updated lateral palatopharyngoplasty at a tertiary university hospital.

Cases with either high or low tongue base collapse during transnasal DISE were excluded from the study because they needed tongue surgery. Still, those with TP interaction without tongue collapse were the subject of study whether this interaction affected the response of palatopharyngoplasty surgery or not, and if this necessitated additional management. In our study, positive TP interaction meant a visual impression of the oropharyngeal tongue pushing the soft palate, causing either its collapse or at least limiting the oral airflow.

We performed the DISE to assess the level and the pattern of obstruction, as the studies comparing it to awake evaluations showed alteration in the surgical management plan in approximately 50% of the patients. 14 The drug propofol was used in our study, as most studies comparing sedation and natural sleep used it or midazolam as a single agent. These drugs had an effect that mimics the critical closing pressure during natural sleep without a significant difference in the AHI. 15 16 17

Other studies 18 19 used simulation models to detect the level of obstruction depending on computational fluid dynamics (CFD). However, in the present study, these tools could not be used their unavailability in our country and the dependence on DISE to detect the level of collapse pattern.

We selected a new Cahali 11 lateral pharyngoplasty with the concept that it can correct all the retropalatal collapse in all dimensions, and also it can stiffen the lateral pharyngeal wall. The study by Cahali proposed that all OSA patients will benefit from the new lateral pharyngoplasty procedure, regardless of the level and pattern of airway obstruction, as neither the thickness of the posterior tonsillar pillar nor the soft TP position are selection factors. His technique for treating sleep apnea has evolved due to factors such as the retropalatal airway being the primary site of obstruction, TP coupling, lateral wall extension, lack of anteroposterior enlargement of the retropalatal area in previous LPs, and residual supine obstruction. His approach was modified to prevent stretching of the pharyngeal mucosa, promote retropalatal expansion, and splint the upper lateral pharyngeal wall with a myomucosal palatopharyngeus flap. 11 Therefore, if the new lateral pharyngoplasty was properly done, any residual collapse should be either at the level of the tongue or larynx, which can be excluded by the routine transnasal DISE, or the palate is being pushed by the tongue, which the transoral DISE can demonstrate.

Elzayat et al. 20 conducted their study on 40 cases undergoing the new Cahali pharyngoplasty operation. They concluded that the technique could be used as a standalone procedure in all OSA patients except those who had a lateral wall collapse at the level of the hypopharynx (LH), high tongue base collapse (TH), laryngeal collapse (L1), and TP interaction.

Our definition of positive TP interaction as absent space between tongue and palate is much different from Friedman or Mallampati scores because interaction usually happens on a dynamic basis, and when revising the static examination data of the 6 positive cases, we found they were not necessarily high grades Friedman or Mallampati.

In our study, the Friedman tongue position (FTP) in the +ve TP interaction group was grade III in 1, II in 3, and I in two patients, meaning that a high Friedman tongue position can't be used as a predictor for positive TP interaction.

According to the European position paper on DISE, 5 the transoral type can be used to assess the degree of tongue retraction and position, as it could be evaluated from the oral cavity and nasopharynx, highlighting a secondary anteroposterior soft palate collapse, due to the tongue position but they did not study its effect on the management plan.

Additionally, Elsobki et al. 10 conducted a cross-sectional study on a total of 30 cases with OSA who underwent DISE according to a new classification system called LwPTL. They demonstrated that 93.3% of the cases presented lateral pharyngeal wall collapse, usually at the level of the velum (73.3%), and 80% presented multilevel collapse. They classified the findings of transoral DISE into TP contact, which occurred between the oropharyngeal tongue and soft palate laterally, and this normally happened in all patients and TP interaction with a visual impression of the tongue pushing the palate back. They mentioned that either positive or negative TP interaction did not affect treatment plans due to lack of standardization.

There is no marked difference in age between both groups. However, +ve TP interaction in females is marked higher than –ve, while BMI in +ve cases is higher than in –ve ones.

In terms of outcomes, the success rate of the studied cases was 61.9% after lateral pharyngoplasty. In addition, there was a marked difference among studied groups in terms of success rate after surgery; with 100% of cases with +ve TP interaction failed versus 27.8% of cases with –ve. The 100% failure does not mean that patients with positive interaction did not gain any improvement but means that their improvement did not meet Sher's success criteria.

Similarly, Elzayat et al. 20 conducted their study on 40 cases with known OSAS undergoing the new Cahali pharyngoplasty operation. They revealed that there were 28 (70%) cases with successful surgery results. Yi et al. 21 also reported a comparable success rate (64.7%) in their study on OSA cases that underwent Z-palatopharyngoplasty (ZP3).

The current study demonstrated a marked difference in minimal oxygen saturation and snoring index among studied groups postoperatively ( *p* = 0.003* and < 0.001*, respectively). In harmony with the current study, Park et al. 22 reported that various parameters such as respiratory disturbance index (RDI), lowest O _2_ saturation, mean O _2_ saturation, oxygen desaturation index, supine AHI, and ESS markedly improved after surgeries in both groups.

In the same line, El Sobki et al. 12 demonstrated that postsurgical STOP-BANG score, AHI, and snoring index were markedly diminished compared with presurgical data. On the contrary, minimal and baseline SpO _2_ were markedly elevated after surgery.

Regarding AHI, the current study demonstrated a marked difference among studied groups in the preoperative score. At the same time, there is a marked difference among studied groups postoperatively, with a higher percentage of improvement among the –ve compared with the +ve TP interaction group. In the same line, Dizdar et al. 23 displayed that the average presurgical AHI of lateral pharyngoplasty cases was 23.5, and the average postsurgical was 11.5. Thus, the average minimal SpO _2_ markedly increased postsurgically in both groups; on the other hand, it was markedly reduced in the lateral pharyngoplasty group compared with the uvulopalatopharyngoplasty (UPPP) ones.

In terms of the ESS, the current study demonstrated a marked difference among studied groups after surgery, without significant difference presurgery. A higher percent of improvement was detected among the –ve TP compared with the +ve TP interaction group.

Regarding the VAS score of snoring, the present study revealed that changes in VAS score after lateral pharyngoplasty were significant in both –ve and +ve TP interaction groups (3.24,4.5) respectively, with *p* = 0.003*.

The study by Li-Ang Lee and Jen-Fang Yu 24 used two procedures to treat snoring, palatal implant and radiofrequency surgery, showing changes in VAS scores following surgeries were significant in the two groups.

The current study displayed a markedly higher average improvement among cases with –ve TP interaction than those with +ve in terms of snoring index, AHI, and ESS. In the same line, Elzayat et al. 20 studied the relationship between the findings of DISE and the operation's success; they reported that transoral DISE can predict operation outcomes while the level of palatal collapse had no significant correlation with the outcome of the operation.

Pang et al. described the modified cautery-assisted palatal stiffening operation (CAPSO) approach, which is performed under local anesthesia. 25 This approach has shown promising outcomes for patients with snoring and mild OSA. 25 In 2009, the updated CAPSO procedure was anterior palatoplasty, focusing on the anterior surface of the soft palate. 26

Barbed anterior palatoplasty is a modified technique, with an identical procedure to the traditional one, with the addition of barbed thread to suspend the suture across multiple mucosa and muscle planes without tying knots. 27 Pang et al. 28 performed a systematic review that has shown that anterior palatoplasty has comparably favorable results to other methods of palatal surgery in adults. The procedure is simple to perform, anatomically sound, and has minimal complications.

There were 2 cases (4.76%) with secondary bleeding and 2 (4.76%) with velopharyngeal insufficiency in terms of complications. In harmony with the current study, Elsobki et al. 12 demonstrated minimal postsurgical complications with no significant long-term morbidity as; secondary bleeding in 2 (4.3%) cases and 1 patient (2.2%) had velopharyngeal insufficiency. Additionally, Park et al. 22 reported that velopharyngeal insufficiency developed in 1 patient, and postsurgical hemorrhage in 4 patients (10%), with stopped immediately in all cases without the need for emergency surgeries.

Palatopharyngeal expansion is usually expected to cause temporary VPI because of the extra effort needed by the velar structures to close the intentionally widened sphincter, with compensation being the rule rather than the exception. 11 Our cases were treated by speech therapy.

According to the results of our study, patients with positive TP interaction do need additional treatment rather than isolated palatopharyngeal expansion, even if their tongues were not collapsing during the routine transnasal DISE. However, further research is needed to evaluate different treatment plans.

The transoral DISE had disadvantages, such as difficulty of introduction, and the need to keep the mouth slightly open which is not physiological. It also provides no data about the nose condition or palatal collapse. The current lack of standardization for its applicability should also be considered.

To the best of our knowledge, this is the first study designed to thoroughly evaluate the effect of positive TP interaction on decision-making in sleep surgery and the possible role of transoral DISE in its evaluation.

One major limitation of this study is its dependence on the only examiner's subjective impression or visual inspection. Future standardization using computer-assisted evaluation may overcome this limitation. Additionally, a larger number of cases and longer observation periods are needed to assess any delayed complications or failures. Another limitation was the absence of a bispectral (BIS) index monitor and target-controlled infusion (TCI) in our institution; however, we always strive to make our DISE data reliable. As such, this last limitation, as well as collapse happening while O _2_ saturation is lower than the nadir in polysomnography, was overlooked.

Transoral DISE is a complementary tool to the conventional transnasal exam, with the objective being to exclude TP interaction to prevent the oral tongue from pushing the soft palate, contributing to its collapse and thus making isolated palatopharyngeal surgery less successful. This was corroborated by our results, despite its small sample size. Other limitations of our work were cases of central retropalatal collapse (which require modified anterior palatoplasty) and lack of positional endoscopy.

## Conclusion

The DISE is considered a vital preoperative assessment tool for OSA cases. We found that the transoral variant can have a valuable role in evaluating and assessing TP interaction and its importance on surgical outcomes. Cases with positive TP interaction show a poor response to isolated palatopharyngeal expansion and need a further treatment plan; in other words, surgery for noncollapsing tongues might be indicated to improve the outcomes.
